# Supplementary material for: Stable transgenerational epigenetic inheritance requires a DNA methylation-sensing circuit
Source: Nat Commun. 2017 Dec 14;8:2124. doi: 10.1038/s41467-017-02219-3 (PMC5730562; doi:10.1038/s41467-017-02219-3)
Supplement: Supplementary file 1 — Supplementary Information [file 41467_2017_2219_MOESM1_ESM.pdf]

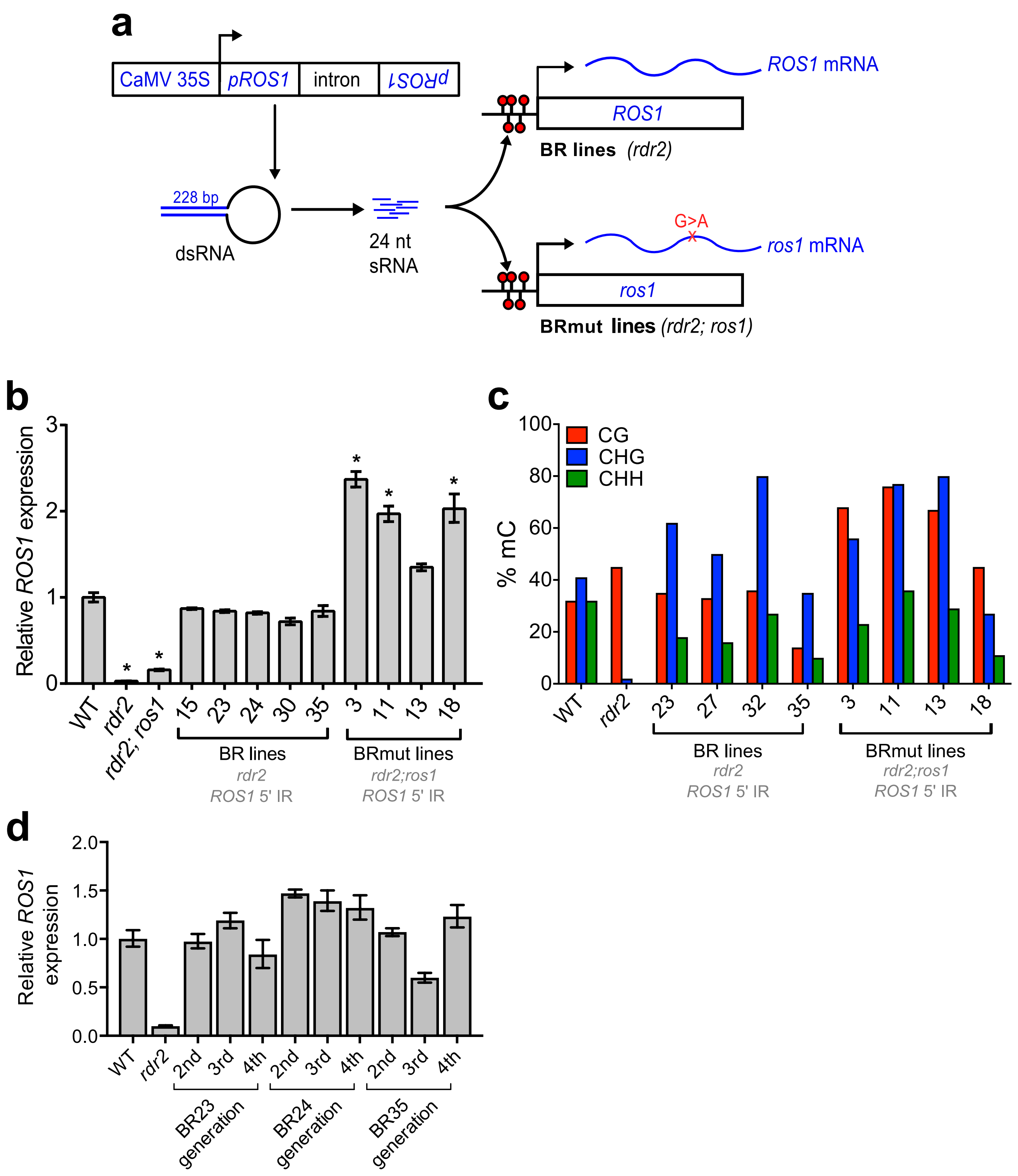

Supplementary Figure 1: Strategy for uncoupling *ROS1* expression from global DNA methylation activity. (**a**) Schematic depicting the inverted repeat (*ROS1* 5' IR) transgene that restores RNA-directed DNA methylation 5' of *ROS1*. The inverted repeat transgene was constitutively expressed in *rdr2* mutants to generate broken rheostat (BR) lines that restore *ROS1* expression to WT levels. The transgene was expressed in *rdr2; ros1* mutants to generate control broken rheostat mutated (BRmut) lines. The mutant *ros1* allele has a missense mutation in the catalytic domain. (**b**) RT-qPCR measurement of *ROS1* transcript abundance in individual plants from second generation BR lines and control lines. Error bars represent standard deviation of three technical replicates. * p = <0.001 in comparison to WT (two-tailed t-test). (**c**) Bisulfite PCR sequencing measurement of DNA methylation 5' of *ROS1* in individual second generation plants from independent BR lines and BRmut lines. Between 12-20 clones were sequenced for each sample. (**d**) RT-qPCR measurement of *ROS1* transcript abundance in second, third and fourth generation plants from 3 independent BR lines. Error bars represent standard deviation of three technical replicates.

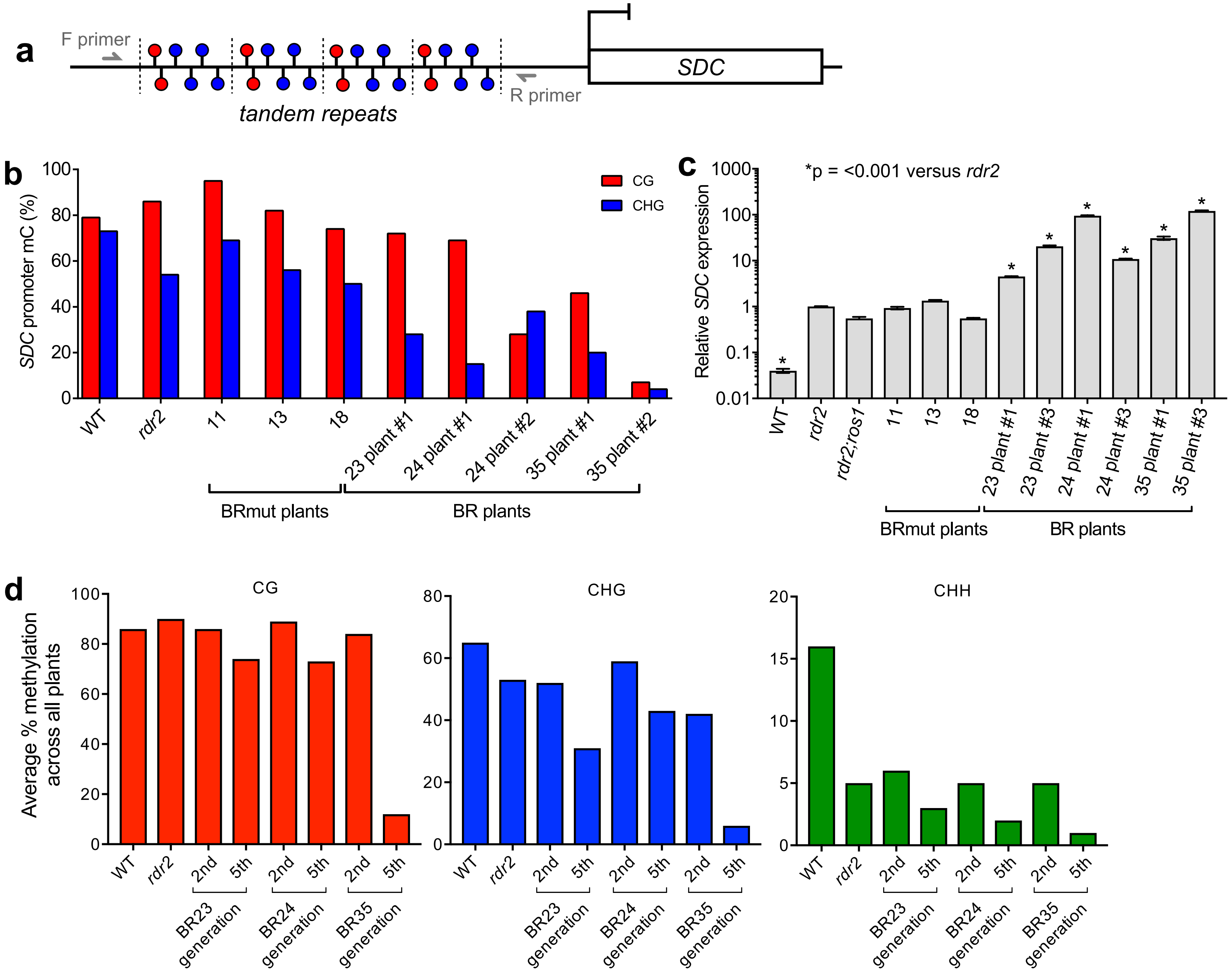

Supplementary Figure 2: *SDC* is hypomethylated and expressed in individual second generation broken rheostat plants. (**a**) Schematic showing methylated *SDC* locus and primers designed for bisulfite PCR. (**b**) Bisulfite PCR measurement of DNA methylation 5' of *SDC* in individual second generation broken rheostat (BR) plants and BRmut plants. At least 24 clones were sequenced for each sample. (**c**) RT-qPCR measurement of relative *SDC* expression in individual second generation BR plants and control plants. Error bars represent standard deviation of three technical replicates. * p = <0.001 in comparison to *rdr2* (two-tailed t-test) (**d**) Bisulfite PCR measurement of average methylation 5' of *SDC* in DNA pooled from ~20 plants from the second and fifth generation of BR lines. Between 24-37 clones were sequenced for each sample. The same data is also represented in Fig. 1e.

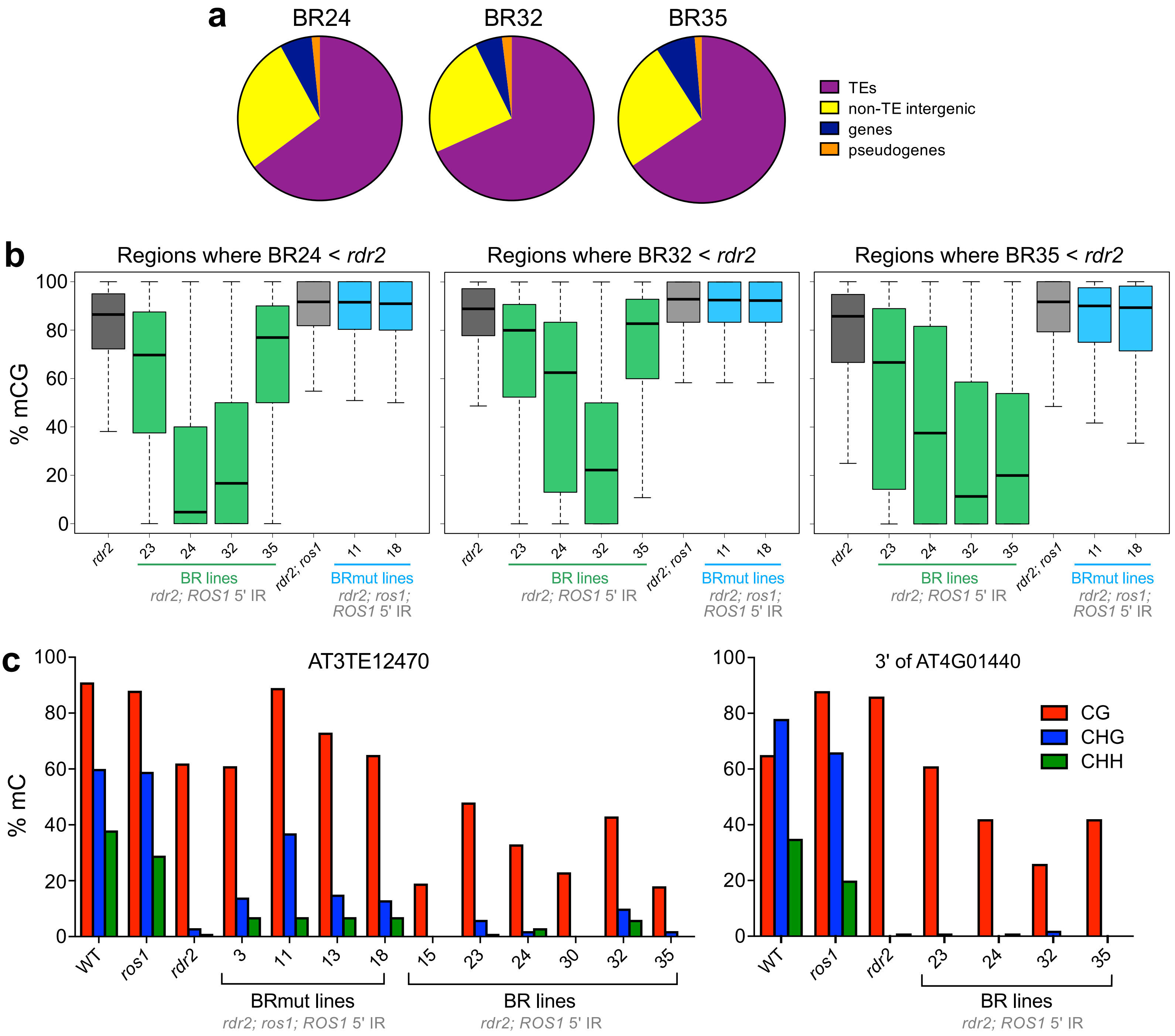

Supplementary Figure 3: Independent broken rheostat lines lose methylation at similar loci. (**a**) Pie charts denoting the proportion of significantly hypomethylated loci (in any sequence context, compared to *rdr2*) that coincide with genes, pseudogenes, transposable elements (TEs) and non-TE intergenic sequences. (**b**) Box and whisker plot of the methylation levels of individual CGs at hypomethylated loci from each BR line. Independent BR lines exhibit reduced methylation compared to *rdr2* at the loci reduced in other lines. (**c**) Bisulfite PCR validation of DNA methylation levels at two loci identified by whole genome bisulfite sequencing to lose DNA methylation in multiple lines. At least 24 clones were sequenced for each sample.

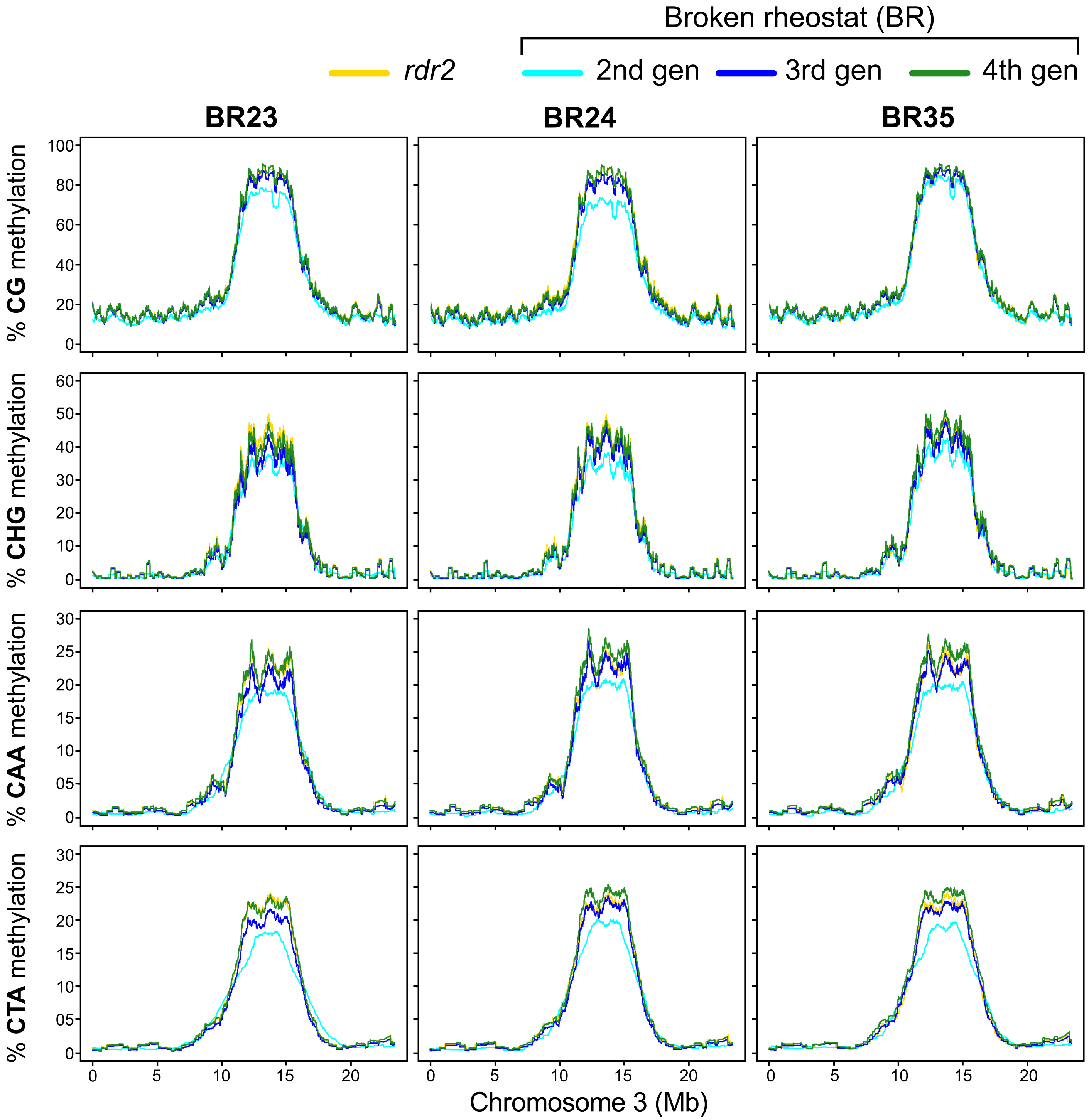

Supplementary Figure 4: Broken rheostat lines gradually regain lost methylation in all sequence contexts. Moving average plots of DNA methylation across chromosome 3 in multiple sequence contexts. Moving average values were calculated across 10 kb windows.

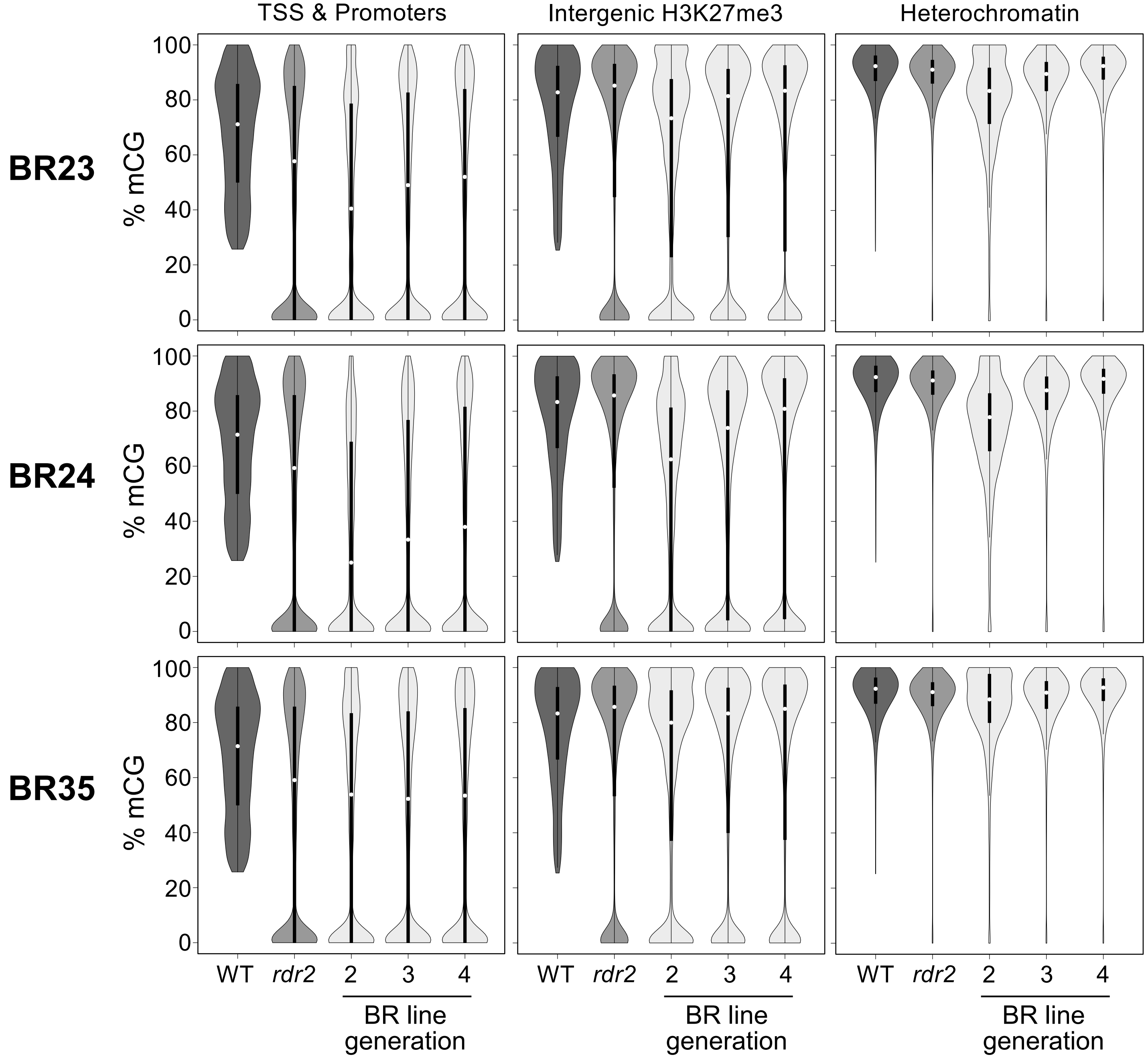

Supplementary Figure 5: Different chromatin states show different dynamic methylation changes over multiple generations. Violin plots of CG methylation levels at different chromatin states. White circles denote median values, black bars denote interquartile ranges. Data is shown for CGs with >= 25% methylation in WT. TSS & promoters represents chromatin states 1 and 2 identified by Sequeira-Mendes et al[1]. Intergenic H3K27me3 represents chromatin states 4 and 5, heterochromatin represents states 8 and 9.

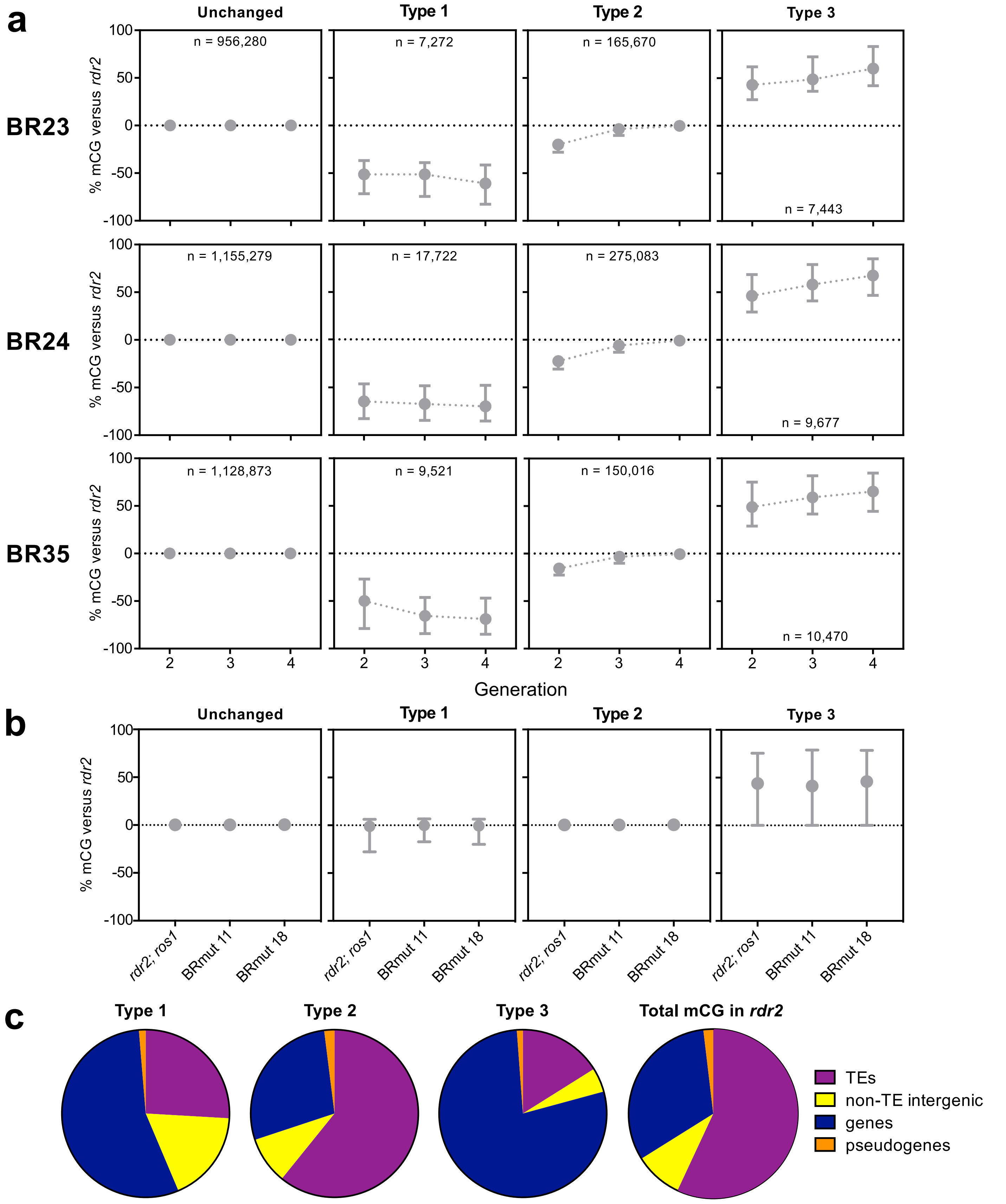

Supplementary Figure 6: K-means clustering of DNA methylation values for individual CGs over multiple generations. (**a**) K-means clustering (k=4) was performed on differential DNA methylation values (compared to *rdr2*) for individual CGs across three generations in three independent broken rheostat (BR) lines. Only cytosines covered by 5 or more reads in all samples were considered. Circles represent median values and error bars represent the interquartile range. n represents the number of CGs in each cluster. (**b**) The methylation level of Type 1, Type 2 and Type 3 CGs in *rdr2; ros1* mutants and BRmut lines. Circles represent median values and error bars represent the interquartile range. (**c**) Pie charts denoting the proportion of Type1, Type 2 and Type 3 CGs that are located within different genomic features.

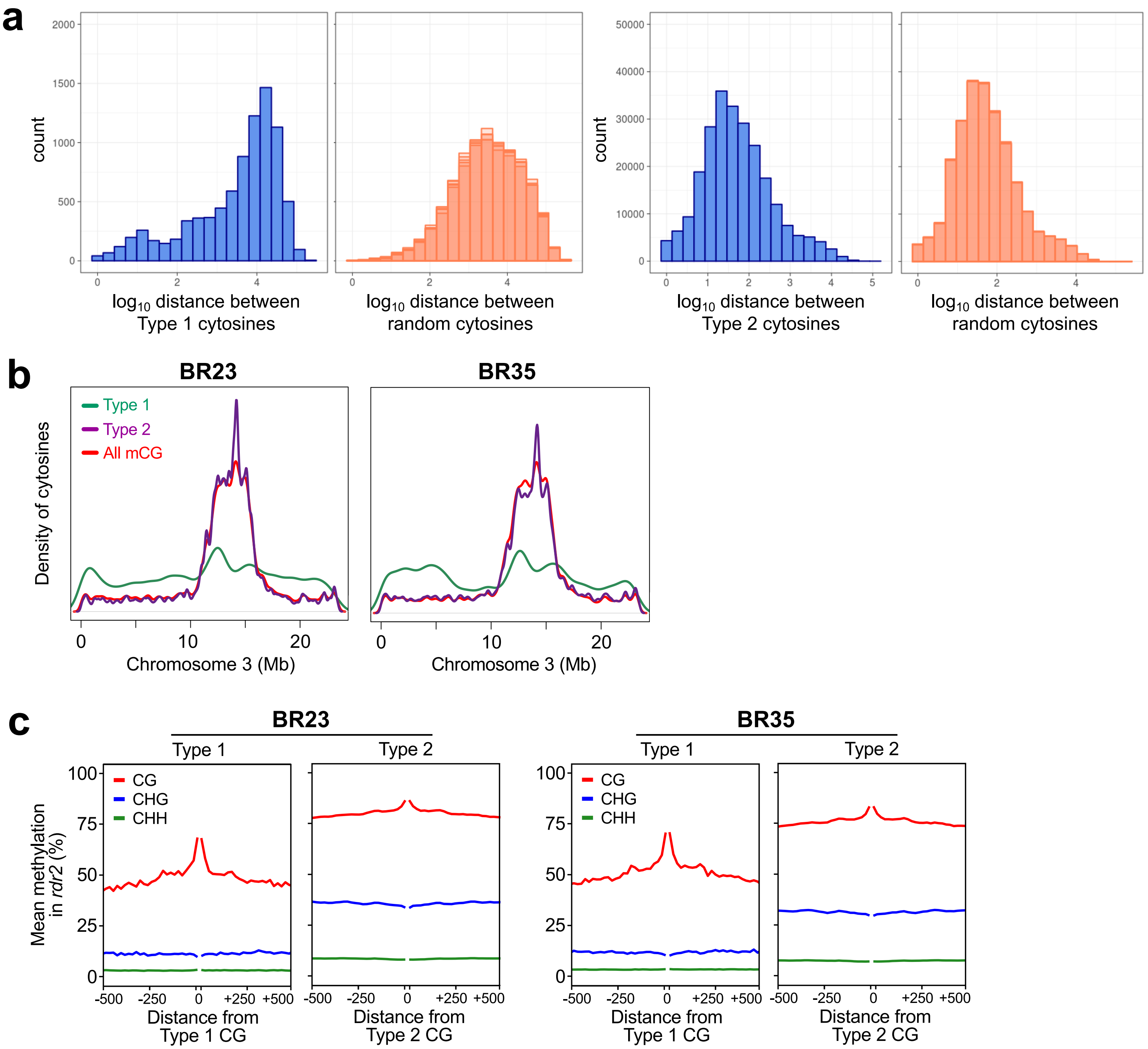

Supplementary Figure 7: Type 1 cytosines are clustered at discrete loci in euchromatic arms. (**a**) The distance between Type 1 or Type 2 CGs to other CGs in the same cluster (in blue) compared to five randomly selected groups of methylated CGs of the same number (in orange). (**b**) Kernel density plots showing the distribution of Type 1 and Type 2 cytosines on chromosome 3 compared to all methylated CGs in *rdr2* for BR lines 23 and 35. (**c**) Average methylation levels of the *rdr2* mutant in sequences proximal to BR23 or BR35 Type 1 and Type 2 CGs. Methylation levels are calculated for sequences up to 500 bp upstream and downstream.

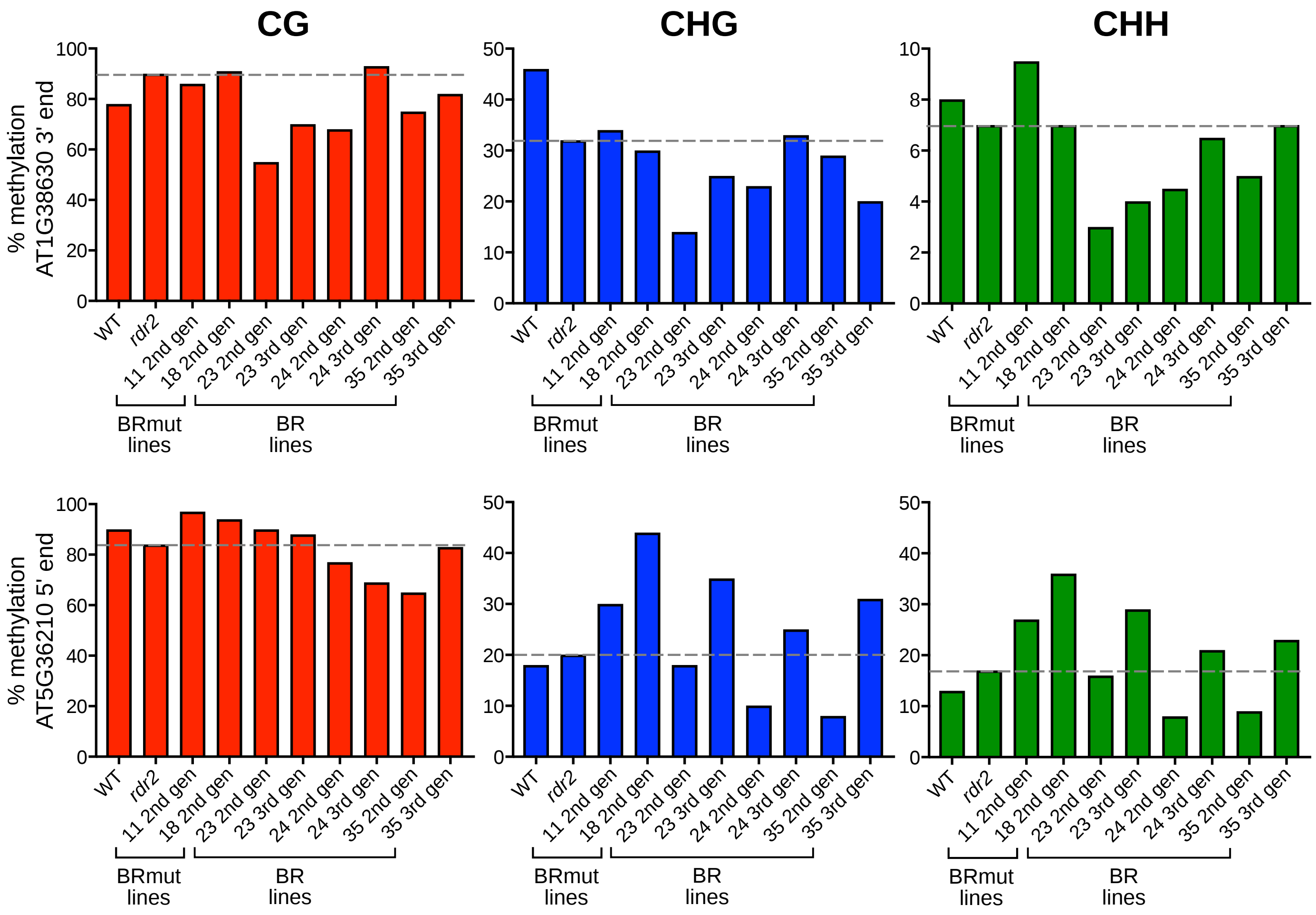

Supplementary Figure 8: Bisulfite PCR validation of Type 2 cytosines in BR lines. Bisulfite PCR measurement of DNA methlyation levels at two loci that regained methylation from the second to third generation in the whole genome bisulfite sequencing data. Bisulfite PCR was performed on DNA from siblings of the individuals used for whole genome bisulfite sequencing. The third generation plants used in this experiment are therefore not progeny of the second generation plants. At least 16 independent clones were sequenced for each sample.

Supplementary Table 1: Statistics on whole genome bisulfite sequencing libraries

Sample	Tissue	Leaf curling phenotype score	cycles	Total reads	Uniquely mapping reads	% Reads uniquely mapping	Unique reads after deduplication	% non-duplicated reads	Conversion rate	mC in CpG context:	mC in CHG context:	mC in CHH context:
Col-0	3-week old rosette	0	100 PE	35,788,973	17,190,627	48.03	10,793,901	62.79	98.3%	39.2%	15.9%	4.5%
rdr2	3-week old rosette	1	100 PE	84,179,767	46,232,091	54.92	21,754,127	47.05	96.7%	34.4%	12.0%	3.1%
ros1	3-week old rosette	1	100 PE	41,061,019	24,046,287	58.56	13,447,384	55.92	98.3%	38.6%	15.1%	4.0%
rdr2; ros1	3-week old rosette	1	100 PE	78,808,019	35,277,213	44.76	20,122,678	57.04	98.7%	35.8%	13.0%	3.3%
BRmut 11*	3-week old rosette	1	100 PE	99,485,220	39,531,759	39.74	21,857,722	55.29	98.7%	35.4%	12.6%	3.1%
BRmut 18*	3-week old rosette	0	100 PE	75,570,327	34,869,357	46.14	19,629,954	56.30	98.4%	36.4%	13.4%	3.4%
BR23*	3-week old rosette	2	100 PE	55,017,230	30,538,823	55.51	13,665,002	44.75	97.5%	36.7%	13.6%	3.7%
BR24*	3-week old rosette	3	100 PE	139,230,332	64,455,143	46.29	31,668,205	49.13	99.4%	31.3%	11.3%	3.0%
BR32*	3-week old rosette	4	100 PE	130,371,424	61,539,987	47.20	30,991,983	50.36	98.8%	29.6%	12.0%	3.1%
BR35*	3-week old rosette	5	100 PE	64,075,533	35,244,745	55.00	16,784,762	47.62	99.0%	36.4%	14.2%	3.4%
BR23 T_2	3-week old mature leaves	1	40/50 PE**	80,706,122	33,580,221	41.61	11,698,237	34.84	99.8%	28.6%	9.1%	2.1%
BR23 T_3	3-week old mature leaves	2	75 PE	61,223,702	31,453,899	51.38	20,379,475	64.79	99.6%	32.7%	9.9%	2.5%
BR23 T_4	3-week old mature leaves	2	100 PE	78,203,515	33,867,703	43.31	19,609,294	57.90	99.5%	34.2%	11.1%	3.0%
BR24 T_2	3-week old mature leaves	2	40/50 PE**	92,583,656	43,009,570	46.45	17,799,330	41.38	99.9%	25.8%	8.3%	2.1%
BR24 T_3	3-week old mature leaves	3	75 PE	63,644,488	34,135,287	53.63	22,771,920	66.71	99.4%	31.2%	10.3%	2.7%
BR24 T_4	3-week old mature leaves	4	100 PE	87,878,709	38,831,174	44.19	21,448,630	55.24	99.4%	33.1%	11.3%	3.2%
BR35 T_2	3-week old mature leaves	3	40/50 PE**	98,997,998	23,397,455	23.63	15,155,753	64.78	98.3%	30.9%	10.2%	2.3%
BR35 T_3	3-week old mature leaves	4	75 PE	59,407,918	27,285,057	45.93	16,956,950	62.15	99.4%	34.9%	11.9%	3.0%
BR35 T_4	3-week old mature leaves	5	100 PE	70,681,260	27,810,950	39.35	15,163,358	54.52	99.5%	36.4%	13.5%	3.5%

*Individual plants from T_4 generation

**Libraries were sequenced twice, once at 40bp paired end and once at 50bp paired end

Supplementary Table 2: Oligonucleotides used in this study

qPCR Primers used in this study

ROS1 qPCR F	CAGGCTTGCTTTTGGAAAGGGTACG
ROS1 qPCR R	GTGCTCTCTCACTCTTAACCATAAGCT
SDC qPCR F	GTAGAAGTCAAGTCCTTGGGAGAT
SDC qPCR R	GAACTCATGAGCCGAAACCGAGA
AT1G58050 qPCR reference F	CCATTCTACTTTTTGGCGGCT
AT1G58050 qPCR reference R	TCAATGGTAACTGATCCACTCTGATG

Bisulfite PCR primers used in this study

ROS1 5' F	GGAGATTTTGTAGAAAAGAATYATT
ROS1 5' R	TCACTRATRCTTCRTTTCTTCTCTT
SDC 5' F	GAAAAAGTTGGAATGGGYTTGGAGAG
SDC 5' R	CTCATTCTRCTTTAAACCTCTATACTTA
AT3TE12470 F	GGAATTTAGGAATTAAGGATTTGTT
AT3TE12470 R	CAACCCAAATATTATRCCAACTC
AT4G01440 3' F	GTATTTTATTGTGTTTTATGTACTTG
AT4G01440 3' R	TTTATCAATCTATCCTATAARTTTTTC
AT1G38630 3' F	GATGTYATGTGTATGATTGAGTTAAGA
AT1G38630 3' R	CTARTTCTTATACTCAATCATACACAT
AT5G36210 5' F	TGAGGATAGATYATTGTAATTGTTGGTTT
AT5G36210 5' R	TCAACAACAACAATRCCATTTATTTACTCA

Supplementary Reference

1. Sequeira-Mendes, J. et al. The functional topography of the *Arabidopsis* genome is organized in a reduced number of linear motifs of chromatin states. *Plant Cell* **26**, 2351–2366 (2014).
